# Systemic sclerosis associated interstitial lung disease - individualized immunosuppressive therapy and course of lung function: results of the EUSTAR group

**DOI:** 10.1186/s13075-018-1517-z

**Published:** 2018-01-30

**Authors:** Sabine Adler, Dörte Huscher, Elise Siegert, Yannick Allanore, László Czirják, Francesco DelGaldo, Christopher P. Denton, Oliver Distler, Marc Frerix, Marco Matucci-Cerinic, Ulf Mueller-Ladner, Ingo-Helmut Tarner, Gabriele Valentini, Ulrich A. Walker, Peter M. Villiger, Gabriela Riemekasten, EUSTAR co-workers on behalf of the DeSScipher project research group within the EUSTAR network

**Affiliations:** 1 Department of Rheumatology, Immunology and Allergology, University Hospital and University of Bern, Freiburgstrasse 4, 3010 Bern, Switzerland; 2 German Rheumatism Research Center, A Leibniz Institute, Berlin, Germany; 3 Department of Rheumatology and Clinical Immunology, Charité University Hospital, Berlin, Germany; 4 Department of Rheumatology A, Descartes University, APHP, Cochin Hospital, Paris, France; 5 Department of Rheumatology and Immunology, University of Pecs, Pecs, Hungary; 6 University of Leeds, Leeds, UK; 7 UCL Division of Medicine, Centre for Rheumatology, Royal Free Hospital, London, UK; 8 Department of Rheumatology, University Hospital Zurich, Zurich, Switzerland; 9 Department of Rheumatology and Clinical Immunology, Osteology and Physical Therapy, Justus-Liebig-University Giessen, Kerckhoff Klinik, Bad Nauheim, Germany; 10 Department Experimental and Clinical Medicine, Division of Rheumatology AOUC, University of Florence, Florence, Italy; 11 Department of Rheumatology, Second University of Naples, Naples, Italy; 12 Department of Rheumatology, University of Basel, Basel, Switzerland; 13 Department of Rheumatology, University Medical Center Schleswig-Holstein, Kiel, Germany

**Keywords:** Systemic sclerosis, Interstitial lung disease, Immunosuppressants, Follow up, Lung function

## Abstract

**Background:**

Interstitial lung disease in systemic sclerosis (SSc-ILD) is a major cause of SSc-related death. Imunosuppressive treatment (IS) is used in patients with SSc for various organ manifestations mainly to ameliorate progression of SSc-ILD. Data on everyday IS prescription patterns and clinical courses of lung function during and after therapy are scarce.

**Methods:**

We analysed patients fulfilling American College of Rheumatology (ACR)/European League against Rheumatism (EULAR) 2013 criteria for SSc-ILD and at least one report of IS. Types of IS, pulmonary function tests (PFT) and PFT courses during IS treatment were evaluated.

**Results:**

EUSTAR contains 3778/11,496 patients with SSc-ILD (33%), with IS in 2681/3,778 (71%). Glucocorticoid (GC) monotherapy was prescribed in 30.6% patients with GC combinations plus cyclophosphamide (CYC) (11.9%), azathioprine (AZA) (9.2%), methotrexate (MTX) (8.7%), or mycophenolate mofetil (MMF) (7.3%). Intensive IS (MMF + GC, CYC or CYC + GC) was started in patients with the worst PFTs and ground glass opacifications on imaging. Patients without IS showed slightly less worsening in forced vital capacity (FVC) when starting with FVC 50–75% or >75%. GC showed negative trends when starting with FVC <50%. Regarding diffusing capacity for carbon monoxide (DLCO), negative DLCO trends were found in patients with MMF.

**Conclusions:**

IS is broadly prescribed in SSc-ILD. Clusters of clinical and functional characteristics guide individualised treatment. Data favour distinguished decision-making, pointing to either watchful waiting and close monitoring in the early stages or start of immunosuppressive treatment in moderately impaired lung function. Advantages of specific IS are difficult to depict due to confounding by indication. Data do not support liberal use of GC in SSc-ILD.

**Electronic supplementary material:**

The online version of this article (10.1186/s13075-018-1517-z) contains supplementary material, which is available to authorized users.

## Background

Interstitial lung disease (ILD) in systemic sclerosis (SSc-ILD) is caused by alveolitis-induced fibrosis of the intra-alveolar tissue, leading to progressive decline in lung function [1]. It is the most frequent cause of SSc-associated death [2]. Current treatment options aim at reducing pulmonary interstitial inflammation in order to prevent progression of fibrosis and consecutive deterioration of lung function.

Cyclophosphamide (CYC) is widely used in the treatment of SSc-ILD, especially in induction therapy as reflected by the European League Against Rheumatism (EULAR) recommendations for SSc-ILD treatment [3]. Unfortunately, the toxicity of CYC makes it unsuitable for long-term use. Furthermore, within the first scleroderma lung study, the effect of CYC waned a few months after cessation [4]. Mycophenolate mofetil (MMF) has been suggested as an alternative for induction and maintenance IS [5] and has been shown to stabilise lung function in two studies [6, 7]. There are recent data from the scleroderma lung study II on the risks and benefits of a 2-year course of MMF versus a 1-year course of oral CYC. Herein, MMF displayed a better safety profile and a 1-year course of CYC improved skin and lung function to a comparable extent [8]. Azathioprine (AZA) reflects the common practice of introducing a steroid-sparing anti-rheumatic agent in patients with idiopathic pulmonary fibrosis yet might be rather harmful [9]. In SSc-ILD, the evidence for AZA is inconclusive [10–12]. Methotrexate (MTX) is recommended for treatment of skin manifestations in early diffuse cutaneous SSc (dcSSc) [13]. Its use in SSc-ILD remains controversial as lung fibrosis is a rare but potentially severe side effect [14] and evidence for anti-fibrotic efficacy in the lungs is lacking. As skin and lung involvement may appear simultaneously, MTX is sometimes prescribed in SSc-ILD. Rituximab (RTX) is among the most frequently used biological agents in SSc with a recent case-control study and an observational study suggesting beneficial effects on lung function [15]. As elevated interleukin-6 (IL-6) in SSc-patients has been associated with higher incidence of progressive pulmonary decline, tocilizumab (TCZ) was recently introduced as a therapeutic strategy within the faSScinate study [16, 17]. This randomised controlled trial demonstrated a benefit from TCZ, with a significantly smaller decline in forced vital capacity (FVC); unfortunately, this effect waned at 48 weeks. Low-dose glucocorticoids (GCs) used to be the standard treatment for SSc-ILD. This is remarkable as GCs have never been shown to improve ILD outcomes and are suspected to dose-dependently increase the risk of SSc renal crisis [18]. GCs are variously prescribed at least initially in combination therapy in severe and progressive ILD [13, 19]. Overall, current evidence does not allow convincing recommendations on the use of IS in ILD. The updated EULAR/European League against Rheumatism Scleroderma Trial and Research (EUSTAR) guidelines will be in line with this conclusion [20].

The EUSTAR database offers a unique opportunity to analyse IS therapy in SSc-ILD. The aims of this study were (1) to analyse current use of IS drugs, (2) to test correlation between drug use and lung function tests and (3) to define specific treatments for defined disease characteristics.

## Methods

We included patients aged ≥ 18 years fulfilling the American College of Rheumatology (ACR) 1980 or ACR/EULAR 2013 classification criteria for SSc [21] with signs of ILD on pulmonary high resolution computed tomography (HRCT) and/or chest x-ray and at least one report on IS.

Data analysis comprised first EUSTAR documentation from 2004 until 6 May 2014. The entire observation period of each patient since initial diagnosis of ILD was considered. In order to receive comprehensive overviews of IS in SSc-ILD we referred to all documented visits at which IS was used. Missing IS information was counted as “never IS” if at least one item from the list of immunosuppressive therapies was answered. Patients with IS therapy (“ever IS”) at any time were compared to patients who had never received IS (“never IS”). For our analysis of “never IS” versus “ever IS” patients were included at the visit when IS was documented for the first time or time of first ILD documentation for “never IS”. For comparison of the features of patients receiving different IS we selected patients with at least one follow up since the documentation of ILD. We then grouped patients according to forced vital capacity (FVC) and diffusing capacity of the lung for carbon monoxide (DLCO) at the initiation of therapy, mimicking the classification by Steen [22] in order to generate three groups with different SSc-ILD severity: “mild” for DLCO >60% and FVC >85%, “moderate” for DLCO 51–60% and FVC 80–85% and “severe” for DLCO <51% and FVC <80%.

Standard EUSTAR documentation comprises current history, past medical history, medications, physical examination including modified Rodnan skin score (mRSS), laboratory results, lung and heart function tests, radiological imaging and capillaroscopy [23]. Disease duration is calculated from the time since first non-Raynaud’s symptom. Yearly follow-up documentation is recommended. As a EUSTAR rule, each participating centre must obtain an ethics vote from their respective ethics committee. Afterwards, participating patients need to sign an individual consent form prior to inclusion into EUSTAR analysis.

### Statistics

Continuous parameters were compared by the Mann-Whitney test, frequencies by the chi^2^ or Fisher exact test; *p* values <0.05 were considered significant. No adjustment for multiple testing was done. Course of lung function under treatment was evaluated by linear regression analysis of change in DLCO and FVC from treatment start to at least one follow-up measurement. Patients were grouped by ranges of starting values (<50%, 50–75% and >75% predicted). Due to small numbers of cases for many treatment combinations, no individual combinations could be considered. Instead, additive and multiplicative effects of single drugs on the overall group trend were tested within each stratum: with additive effects indicating patients were taking that drug over the same overall time trend, but at a higher or lower level, meaning better or worse initial lung function, but afterwards the same course of DLCO or FVC; and with multiplicative effects signalling a steeper slope of the trend for patients using that drug, meaning either a better or worse course than the overall trend. We adjusted for the potential confounders of sex, age, extent of skin involvement, disease duration and initial DLCO or FVC values, respectively. Statistical analyses were performed using IBM SPSS Statistics, version 19.

## Results

### Patients on IS have more severe and active ILD compared to those without IS

Epidemiological data are shown in Table 1. Overall, IS was used in 2681/3778 (71%) patients with SSc-ILD, but only in 39.8% of patients with SSc without ILD (*p <* 0.05, data not shown).

**Table 1:** Characteristics of patients with SSc-ILD never or ever using immunosuppressive therapy

	Total	Never used IS therapy	Ever used IS therapy	*P* value
Number of patients	3778	1097 (29%)	2681 (71%)	
Age (mean, SD)	55.5 ± 13.4	59.0 ± 13.6	54.0 ± 13.0	<0.001
Female	83.6%	86.5%	82.4%	0.002
BMI (mean, SD) (*n* = 1901)	24.6 ± 4.7	24.5 ± 5.1	24.7 ± 4.6	n.s.
Duration of SSc, years (mean, SD)	8.5 ± 7.9	10.8 ± 9.2	7.6 ± 7.1	<0.001
(median (IQR))	6.2 (2.8; 11.9)	8.4 (4.3; 15.0)	5.4 (2.4; 10.8)	
mRSS (*n* = 3515)				
(mean, SD)	10.6 ± 8.7	8.9 ± 7.6	11.3 ± 9.0	<0.001
(median (IQR))	8.0 (4.0; 17.0)	7.0 (4.0; 17.0)	9.0 (4.0; 17.0)	
Extent of skin involvement (*n* = 3713)				
diffuse	44.4%	29.4%	50.3%	
limited	46.9%	60.9%	41.3%	<0.001
sclerodactyly only	7.5%	7.4%	7.6%	
none	1.2%	2.4%	0.8%	
Present scleroderma pattern (n = 1081)	92.6%	92.3%	92.7%	n.s.
active	40.7%	41.5%	40.4%	
early	21.1%	25.3%	19.4%	0.076
late	38.2%	33.2%	40.2%	
SSc activity index ≥3 (*n* = 3557)	20.0%	12.8%	22.9%	<0.001
DLCO, % predicted (mean, SD) (*n* = 2909)	62.0 ± 20.2	67.4 ± 19.8	59.9 ± 20.0	<0.001
FVC, % predicted (mean, SD) (*n* = 2239)	87.5 ± 21.8	94.9 ± 20.9	84.4 ± 21.5	<0.001
FVC:DLCO ratio (*n* = 2072)	1.5 ± 0.5	1.5 ± 0.5	1.5 ± 0.5	n.s.
FEV-1, % predicted (mean, SD) (*n* = 2239)	86.2 ± 19.9	90.7 ± 19.0	84.2 ± 20.0	<0.001
TLC, % predicted (mean, SD) (*n* = 2239)	85.0 ± 20.5	90.6 ± 20.1	82.6 ± 20.2	<0.001
History (*n* = 3755)				
worsening of skin	18.2%	12.5%	20.6%	<0.001
worsening of fingers	22.7%	20.7%	23.6%	n.s.
esophageal symptoms	66.2%	65.0%	66.6%	n.s.
stomach symptoms	25.9%	22.1%	27.5%	<0.001
intestinal symptoms	24.9%	24.2%	25.2%	n.s.
arterial hypertension	23.2%	23.7%	23.0%	n.s.
renal crisis	2.0%	1.7%	2.1%	n.s.
dyspnoea	17.3%	12.2%	19.4%	<0.001
worsening of cardiopulmonary manifestations	19.1%	14.8%	20.9%	<0.001
palpitations	27.9%	23.8%	29.5%	<0.001
Raynaud’s present	96.7%	96.1%	97.0%	n.s.
NYHA class (*n* = 2426)				
I	44.0%	49.9%	41.6%	
II	38.7%	37.8%	39.0%	<0.001
III	15.2%	10.1%	17.3%	
IV	2.1%	2.1%	2.1%	
Laboratory measures (*n* = 1346 –,648)				
ANA+	94.9%	95.6%	94.6%	n.s.
ACA+	21.9%	38.6%	15.1%	<0.001
SCL70+	48.8%	36.4%	53.8%	<0.001
U1 RNP+	6.4%	2.9%	7.9%	<0.001
RNA+	4.5%	4.1%	4.7%	n.s.
PM-Scl+	4.5%	3.7%	4.9%	n.s.
CRP elevation	27.5%	18.5%	31.3%	<0.001
CK elevation	9.3%	5.8%	10.7%	<0.001
Proteinuria	6.4%	5.7%	6.7%	n.s.
Hypocomplementemia	6.0%	4.9%	6.4%	n.s.
ESR mm/h (mean, SD)	25.8 ± 20.7	23.7 ± 17.6	26.7 ± 21.7	0.048
Conduction blocks (*n* = 3451)	14.1%	12.8%	14.6%	n.s.
Pulmonary hypertension (*n* = 3451)	23.2%	22.7%	23.3%	n.s.
Diastolic function abnormal (*n* = 3363)	24.3%	21.1%	25.5%	0.008
Pericardial effusion (*n* = 2227)	12.6%	13.2%	12.4%	n.s.
Ground glass opacification (*n* = 2014)	41.1%	30.3%	45.5%	<0.001
PFT restrictive defect (*n* = 3457)	45.9%	35.6%	49.9%	<0.001
Echo				
LVEF (%) (*n* = 2095)	61.4 ± 6.2	62 ± 6.6	61.8 ± 6.5	0.005
PAPsys (mmHg) (*n* = 1835)	33 ± 14.9	32.4 ± 12.7	32.6 ± 13.4	n.s.
Right heart catheter				
RVSP (mmHg) (*n* = 96)	46.8 ± 21.9	40.5 ± 17.9	42.5 ± 19.3	n.s.
PAPmean (mmHg) (*n* = 146)	36.2 ± 15.4	28.5 ± 12.0	30.8 ± 13.5	0.003
PVR (dyn · sec · cm-5) (*n* = 94)	541.5 ± 498	213.5 ± 258.1	328.6 ± 391.2	0.001
PWP (mmHg) (*n* = 113)	12.1 ± 7.0	12.2 ± 9.6	12.2 ± 9.0	n.s.
CI (l/min/m2) (*n* = 100)	2.8 ± 0.6	3.3 ± 1.1	3.1 ± 1.0	0.021
6 MWD				
6 MWD (m) (*n* = 551)	444.9 ± 129.6	421.3 ± 123.8	428.1 ± 125.9	0.021
O2 saturation at rest (*n* = 457)	96 ± 6.3	95.9 ± 4.3	96.0 ± 5.0	n.s.
O2 saturation at exercise (*n* = 389)	92.9 ± 8.0	92.2 ± 8.0	92.4 ± 8.0	n.s.

*BMI* body mass index, *mRSS* modified Rodnan skin score, *DLCO* diffusing capacity of the lung for carbon monoxide, *FVC* forced vital capacity, *FEV-1* forced expiratory volume in one second, *TLC* total lung capacity, *NYHA* New York Heart Association, *ANA* anti-nuclear antibodies, *ACA* anti-centromere antibodies, *SCL70* anti-topoisomerase I antibody, *U1 RNP* U1-small nuclear ribonucloprotein particle, *RNA* ribonucleic acid antibody, *PM SCL* polymyositis scleroderma antibody, *CRP* C-reactive protein, *CK* creatin kinase, *ESR* erythrocyte sedimentation rate, *PFT* pulmonary function test, *LVEF* left ventricular ejection fraction, *PAPsys* systolic pulmonary arterial pressure, *RVSP* right ventricular systolic pressure, *PAPmean* mean pulmonary arterial pressure, *PVR* pulmonary vascular resistance, *PWP* pulmonary wedge pressure, *CI* cardiac index, *6 MWD* 6 minute walk distance, *n.s.* not significant

### IS is used in a wide variety of monotherapy or combination therapy

**Fig. 1:**
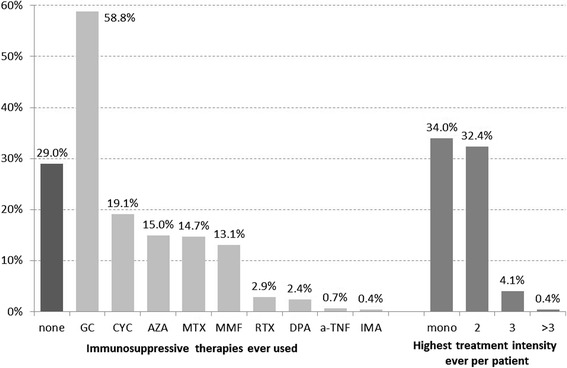
Frequencies of immunosuppressants ever used and highest therapy combination ever used per patient. *GC* glucocorticoids, *CYC* cyclophosphamide, *AZA* azathioprine, *MTX* methotrexate, *MMF* mycophenolate mofetil, *RTX* rituximab, *DPA* D-penicillamine, *a-TNF* anti-tumour necrosis factor, *IMA* imatinib

Frequencies of immunosuppressants ever used and highest therapy combinations ever used per patient are shown in Fig. 1. Of the patients taking GC therapy, the average prednisone dosage was > 10 mg/day in 17%, and > 20 mg/day in 5.3% of patients.

**Fig. 2:**
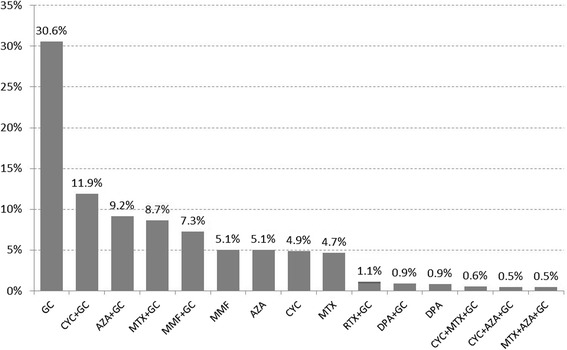
Monotherapies (Mono) and combinations of immunosuppressants ever used, percentages are based on the number of patients. Treatment regimens with frequencies <0.5% were omitted. *GC* glucocorticoids, *CYC* cyclophosphamide, *AZA* azathioprine, *MTX* methotrexate, *MMF* mycophenolate mofetil, *RTX* rituximab, *DPA* D-penicillamine, *a-TNF* anti-tumour necrosis factor, *IMA* imatinib

Individual treatment regimens are shown in Fig. 2, with more than 3 immunosuppressive drugs being exceptions (*n* = 17 patients, not shown).

### Intensive IS is reserved for patients with severe and active ILD

Patient characteristics at the start of the most frequent monotherapy and combination therapy are described in Table 2.

**Table 2:** Characteristics of patients with dSSc or lSSc at the start of specific therapy

	Never used IS		AZA		CYC		MMF		MTX		GC		AZA + GC		CYC + GC		MMF + GC		MTX + GC	
	*n*	%	*n*	%	*n*	%	*n*	%	*n*	%	*n*	%	*n*	%	*n*	%	*n*	%	*n*	%
Sex																				
male	103	14.4	14	10.6	18	24.0	22	20.4	18	17.8	108	15.2	42	18.5	35	21.0	47	24.2	29	14.9
female	612	85.6	118	89.4	57	76.0	86	79.6	83	82.2	604	84.8	185	81.5	132	79.0	147	75.8	165	85.1
total	715	100.0	132	100.0	75	100.0	108	100.0	101	100.0	712	100.0	227	100.0	167	100.0	194	100.0	194	100.0
Extent of skin involvement																				
diffuse	278	38.9	74	56.1	46	61.3	79	73.1	71	70.3	389	54.6	132	58.1	96	57.5	137	70.6	119	61.3
limited	437	61.1	58	43.9	29	38.7	29	26.9	30	29.7	323	45.4	95	41.9	71	42.5	57	29.4	75	38.7
total	715	100.0	132	100.0	75	100.0	108	100.0	101	100.0	712	100.0	227	100.0	167	100.0	194	100.0	194	100.0
NYHA																				
I	200	53.2	63	50.0	21	30.9	43	45.3	47	52.2	260	39.4	80	37.6	45	27.8	64	36.4	82	46.6
II	128	34.0	50	39.7	26	38.2	34	35.8	34	37.8	254	38.5	82	38.5	69	42.6	66	37.5	78	44.3
III	40	10.6	10	7.9	19	27.9	17	17.9	9	10.0	118	17.9	39	18.3	42	25.9	38	21.6	15	8.5
IV	8	2.1	3	2.4	2	2.9	1	1.1	0	.0	28	4.2	12	5.6	6	3.7	8	4.5	1	.6
total	376	100.0	126	100.0	68	100.0	95	100.0	90	100.0	660	100.0	213	100.0	162	100.0	176	100.0	176	100.0
	of N	%	of N	%	of N	%	of N	%	of N	%	of N	%	of N	%	of N	%	of N	%	of n	%
ACA+	662	36.7	101	12.9	56	12.5	89	11.2	81	9.9	614	18.9	191	9.4	153	5.9	149	9.4	165	17.0
SCL70+	665	38.2	106	64.2	59	62.7	92	56.5	85	64.7	617	53.3	197	57.4	155	64.5	157	64.3	167	59.3
Pulmonary hypertension	657	22.5	111	13.5	55	34.5	93	23.7	83	14.5	588	23.1	195	25.1	151	29.1	156	28.8	166	16.3
Ground glass opacification	315	24.4	67	53.7	51	54.9	68	61.8	49	42.9	435	36.3	138	42.0	121	59.5	117	48.7	105	38.1
PFT restrictive defect	666	36.6	102	48.0	56	62.5	78	60.3	82	37.8	574	42.9	191	50.8	151	59.6	144	61.8	161	41.0
	mean	std	mean	std	Mean	std	mean	std	mean	std	mean	std	mean	std	mean	std	mean	std	mean	std
Age (years)	58.6	14.0	56.5	11.5	55.7	12.4	55.4	12.4	54.8	12.0	58.9	12.7	54.1	12.3	53.9	13.1	53.7	12.7	56.9	12.9
Body mass index	24.4	5.0	25.3	4.9	25.3	4.1	25.1	5.1	25.0	5.0	25.0	4.7	25.3	4.8	24.5	3.5	26.2	5.1	24.7	5.4
Disease duration (years)	10.4	8.5	10.4	7.3	9.4	6.6	10.6	7.3	8.2	4.8	11.9	7.6	10.1	6.8	8.6	6.2	9.8	7.0	10.0	7.4
mRSS	9.6	7.6	6.8	5.7	11.6	7.4	9.9	8.2	10.4	8.5	8.9	8.0	8.2	7.1	10.2	8.1	10.3	8.4	11.0	7.9
DLCO (% pred.)	68.0	19.7	59.8	17.2	46.3	18.8	55.7	16.7	64.1	17.8	57.3	19.8	55.5	19.6	49.6	18.2	50.7	16.4	60.0	18.2
FVC (% pred.)	95.7	20.6	86.6	18.9	73.6	20.4	81.8	20.9	89.9	19.6	85.0	22.6	79.9	19.4	77.3	20.3	77.0	22.1	85.8	20.2
FVC:DLCO ratio	1.5	.5	1.6	.5	1.8	.6	1.7	.7	1.4	.4	1.6	.5	1.6	.5	1.7	.6	1.7	.6	1.5	.5
FEV-1 (% pred.)	93.5	18.8	84.0	17.1	77.5	21.4	80.5	18.6	91.2	17.7	84.9	21.8	81.4	18.4	79.9	21.9	76.8	22.5	85.3	18.8
TLC (% pred.)	89.1	20.5	81.2	18.8	70.0	16.5	75.9	18.1	92.5	18.5	80.0	19.3	75.4	21.1	72.5	16.7	74.0	17.8	84.9	20.1

Only therapy regimens with frequencies of at least 5% are displayed (for patients on immunosuppressive therapy, only therapy episodes started during follow up were included to exclude possibly long-lasting therapy episodes documented at baseline). *dsSSc* diffuse cutaneous systemic sclerosis, *lSSc* limited systemic sclerosis, *IS* immunosuppression, *AZA* azathioprine, *CYC* cyclophosphamide, *MMF* mycophenolate mofetil, *MTX* methotrexate, *GC* glucocorticoid, *NYHA* New York Heart Association, *ACA* anti-centromere antibodies, *SCL70* anti-topoisomerase I antibody, *PFT* pulmonary function test, *mRSS* modified Rodnan skin score, *DLCO* diffusing capacity of the lung for carbon monoxide, *FVC* forced vital capacity, *FEV-1* forced expiratory volume in one second, *TLC* total lung capacity, *pred.* predicted, *N* number of patients within this specific group of medication

Compared to patients in the never IS group, patients receiving GC monotherapy had significantly higher prevalence of SSc-related organ complications except for pulmonary hypertension and renal crisis. Within the “ever IS” group, patients receiving GC monotherapy were the oldest and had the longest disease duration. Patients who took MTX were only slightly different from patients in the never IS group. Patients who took MTX/GC had significantly worse DLCO, forced expiratory volume in one second (FEV-1) and modified Rodnan skin score (mRSS), indicating more severe disease and possibly also concomitant obstructive pulmonary disease. Patients who took AZA had significant impairment in FVC and DLCO, but no differences in New York Heart Association (NYHA) class. Interestingly, they had lower mRSS values than the never IS group. AZA/GC was used in patients with a more prominent reduction in DLCO and FVC values and with more patients in NYHA III and IV than in AZA monotherapy. Patients who took MMF had severe impairment of FVC and DLCO, which was even more pronounced when GC was added to MMF. Values for DLCO and FVC were lower and severe NYHA classification more frequent than in MMF monotherapy. Patients who took CYC had the most severely impaired lung function and highest rate of restrictive lung disease. Patients receiving MTX monotherapy had the best values for DLCO, FVC (*p* < 0.001) and total lung capacity (TLC), lowest prevalence of pulmonary hypertension (*p* < 0.05) and shortest disease duration. In contrast, patients who took CYC monotherapy had worst impairment in lung function and the highest rates of ground glass opacifications on imaging, plus the most severe skin fibrosis and highest mRSS values.

### A cluster of lung function parameters is associated with specific choices of IS

**Fig. 3:**
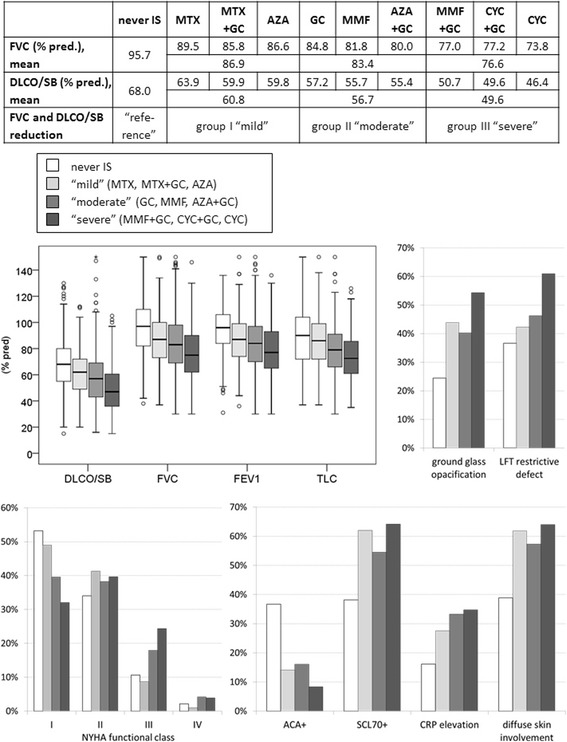
Patients grouped according to severity of lung function combined with the respective immunosuppression (IS) and clinical parameters. *never IS* patients who had never taken immunosuppressant drugs, *FVC* forced vital capacity, *DLCO* diffusing capacity of the lung for carbon monoxide, *SB* single breath, *MTX* methotrexate, *GC* glucocorticoid, *AZA* azathioprine, *MMF* mycophenolate mofetil, *CYC* cyclophosphamide, *PFT* pulmonary function test, *NYHA* New York Heart Association, *ACA* anti-centromere antibodies, *SCL70* anti-topoisomerase I antibody, *CRP* C-reactive protein

Sorting different therapy arms by average impairment in FVC and DLCO revealed clusters of ranges of lung function. Consequently, we grouped patients based on mean FVC and DLCO according to the classification of Steen [22]. Group I (mild impairment) had FVC of 86.9% and DLCO of 60.8%; group II (moderate impairment) had FVC of 83.4% and DLCO of 56.7%; and group III (severe impairment) had FVC of 76.6% and DLCO of 49.6%. Next we assessed whether other parameters were associated with specific choices of IS (Fig. 3).

Patients in group III had the worst FVC and DLCO, highest mRSS values, worst NYHA class and the highest rates of ground glass opacifications and restrictive defects. Compared to patients in the never IS group, patients in all three groups had significantly worse FVC, DLCO and TLC and more frequent ground glass opacifications. Patients in groups II and III had more severe NYHA class (both *p* < 0.001).

Compared to patients in the never IS group, the rate of ground glass opacification rates (24.4%) was twice that in groups I and II (43.9% and 40.2%, respectively), and even more often in group III (54.3%, all treatment groups *p* < 0.001). The same trend was seen in the frequency of restrictive defects with the lowest rates in patients in the never IS group (36%) and an increasing frequency of 46.3% in group II and 61.0% in group III (both *p* < 0.001).

### Specific types of IS display minimal influence on the course of lung function

**Fig. 4:**
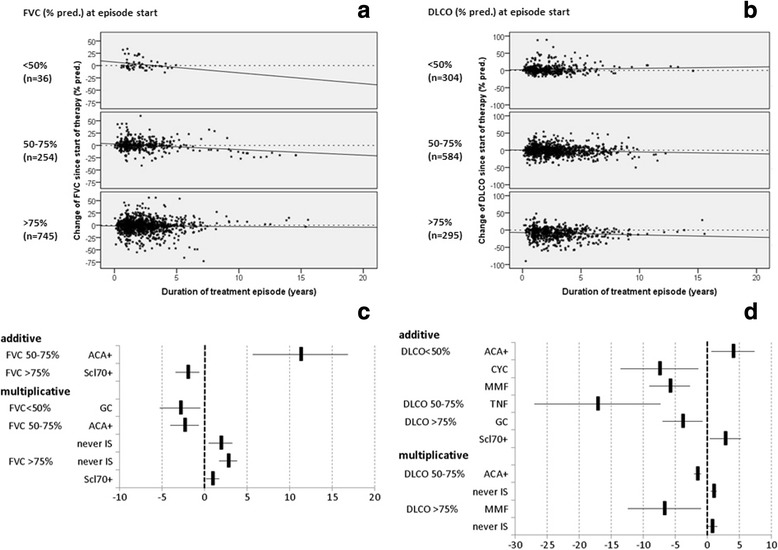
Change in lung function over all patients distinguished in three categories of values at the start of specific therapy (or at baseline for patients who never took immunosuppressant therapy (never IS)) assessed by forced vital capacity (FVC) (**a**) and diffusing capacity of the lung for carbon monoxide (DLCO) (**b**). Effects of different therapies on change in FVC (**c**) and change in DLCO (**d**) compared to the overall trend within these three categories, adjusted for differences in sex, age, disease duration, extent of skin involvement and initial FVC or DLCO value, respectively. Additive effects indicate the same slope shifted to a higher (+) or lower (-) level; positive multiplicative effects indicate a steeper rising or less declining slope; negative multiplicative effects indicate a stronger declining slope. *CYC* cyclophosphamide, *MMF* mycophenolate mofetil, *TNF* tumor necrosis factor inhibitor, *GC* glucocorticoid, *MTX* methotrexate, *AZA* azathioprine, *IS* immunosuppression

Follow-up documentation ranging from 1 month to 13 years was available in 73.6% of patients with SSc-ILD. Change in lung function over time was analysed in the respective subgroups of patients with <50%, 50–75% and >75% of FVC or DLCO predicted at treatment initiation and are shown in Fig. 4.

The group starting with < 50% of predicted FVC had the steepest decline in FVC (Fig. 4a). Here, GCs had negative multiplicative effects (Fig. 4c), thus there was even worse deterioration. In comparison, the group starting between 50 and 75% of predicted FVC had a less steep decline (Fig. 4a), with a positive additive effect in ACA-positive patients (Fig. 4b), but at the same time a negative multiplicative effect, meaning they started at higher FVC levels but had a steeper decline. Here, in patients in the never IS group a positive multiplicative effect was seen, indicating a less steep decline. The group starting with > 75% of predicted FVC had only a slight decline in FVC (Fig. 4a, b) with a negative additive effect in Scl70-positive patients but at the same time a positive multiplicative effect (Fig. 4c), meaning they started at lower FVC levels but had a flatter rate of decline. Here again, there were positive multiplicative effects in patients in the never IS group or in those receiving GCs, indicating less or no decline or even slight improvement.

Within the group starting with < 50% of predicted DLCO the overall course was represented by a slightly improving slope (Fig. 4b). Compared to that general trend, CYC and MMF had negative additive effects, meaning their course was following the same slope, but on a lower level, while ACA positivity had a positive additive effect, hence the course was on a higher level (Fig. 4d). In patients starting with DLCO values between 50 and 75% of predicted the overall trend was of slight deterioration (Fig. 4b), with a negative additive effect of TNF inhibitors, again meaning it already started at a lower level but had the same gradient of deterioration. Here, ACA had a slightly negative multiplicative effect, meaning DLCO courses had a steeper decrease than the general trend, while in patients in the never IS group a positive multiplicative effect was seen, meaning a flatter decrease (Fig. 4b, d), with a positive additive effect of Scl70 positivity and a negative additive effect of GCs. There was a negative multiplicative effect of MMF, meaning an even steeper decrease than the general trend, while there was a positive multiplicative effect in patients in the never IS group (Fig. 4d), meaning less decline or even slight improvement compared to the overall trend.

Adjusted for potential confounders and initial FVC or DLCO value no other medications than GCs, MMF, or “never IS” showed multiplicative effects on the course of lung function divergent from the general trend of the entire patient population. CYC and TNF inhibitors had only additive effects, pointing toward lower initial DLCO or FVC values in these patients, but no differing time trends compared to patients on other treatments.

## Discussion

Our analysis of observational data describes current IS strategies in patients with SSc-ILD from the EUSTAR cohort. It shows clusters of clinical characteristics correlated with IS choices and identifies factors that might influence future IS decisions.

A large proportion of patients with SSc-ILD did not receive IS despite having dcSSc (34% of patients), active scleroderma pattern on nail fold capillaroscopy (40% of patients) and Valentini disease activity index (VDAI) ≥3 (12% of patients). On average these patients have longer disease duration and show fewer signs of alveolitis on HRCT. These characteristics may suggest that the greatest decline in lung function has already happened and stabilisation of lung function in the absence of active inflammation is expected without any further IS medication [22], or they might represent patients with overall benign disease courses. In our analysis, positive trends in lung function over time - especially in patients starting with 50–75% of predicted FVC - support this notion. It contrasts with a scleroderma lung study showing a mild 12-month decline in FVC of 4.2%, and in DLCO of 8.2%, irrespective of disease duration [1]. On the other hand our data are in agreement with a study documenting that FVC values within the first 3 years after disease onset strongly predict SSc-ILD outcome [24].

GCs were used most frequently in 58% of the patients in high proportions and even at dosages >10 mg/day and >20 mg/day. This comes as a surprise and has to be questioned, as the effect of GCs on lung function was marginal at best and only slightly positive in patients with > 75% of predicted FVC who might as well continue without any IS at all. Furthermore, it is well-established that this treatment regimen is associated with higher rates of infection and scleroderma renal crisis [25]. Of note, patients receive combinations of GC with MTX, AZA, MMF or CYC regardless of lung function parameters or NYHA class. Collectively our data show that combining GCs with another IS therapy reflects the standard of care in many centres worldwide.

The second most frequently used therapy was CYC, as recommended by the EUSTAR guidelines for patients with severe and progressive SSc-ILD. Two meta-analyses failed to show a significant benefit of CYC on SSc-ILD on lung function tests [26]. However, a statistically non-relevant improvement might still represent a patient-relevant effect on quality of life as reflected by the Short Form-36 (SF-36) data evaluated within the first scleroderma lung study [4]. In our analysis, patients treated with CYC monotherapy or CYC/GC started with the worst DLCO values, worst NYHA class, highest frequencies of restrictive defects and PH, and the highest mRSS. More than 60% of these patients showed signs of active inflammation reflected by high ESR, CRP elevation or ground glass opacifications on HRCT. Stratification of these patients by their starting values did not result in differences in the slope of DLCO or FVC values compared to all other patients. Interestingly, Becker et al. describe the highest SSc-ILD response rates assessed by FVC and DLCO in patients with low FVC values prior to CYC therapy [27] indicating a potential for reversal of fibrosis.

MMF, often regarded as potential maintenance therapy in SSc-ILD, was used as monotherapy in moderate or - combined with GC - severe lung impairment, in our analysis. A prospective open-label trial on MMF describes early and significant improvement in DLCO, non-significant improvement in FVC and reduction in ground glass opacifications in five patients with disease duration between 1.5 and 3 years [28]. A meta-analysis argues along these lines, suggesting that MMF may stabilise lung function [29]; however, the superiority of MMF compared to CYC was not verified [7]. The very recent randomized controlled, double-blind, parallel group trial comparing MMF with oral CYC shows significant improvement in pre-specified measures of lung function over 2 years. It did not reach its primary endpoint, i.e. MMF to be more effective than CYC; however, MMF had a better side-effect profile [8].

An uncontrolled study of AZA maintenance therapy after 1-year induction with CYC showed stabilising effects of AZA in SSc-ILD, but involved only 13 patients [30]. Retrospective data on 36 patients with SSc-ILD comparing oral CYC with AZA shows significant effects of AZA on DLCO and FVC, yet no effects of CYC [12]. Our data cannot confirm this as AZA had only a very slightly positive effect on DLCO in patients starting with DLCO >75%, thus being almost equal to the effect of “never IS”.

Studies analyzing the effects of MTX on SSc-ILD are rare. This might be due to the fact that ILD is one of the possible side effects of MTX and hence physicians might be hesitant to prescribe it to patients with SSc-ILD. Indeed, there is one study showing no effects of MTX on lung function despite trends towards positive effects on the mRSS [31]. However, this study was small (*n* = 29) and covered a relatively short timeframe (24 weeks). A study with 11 patients taking MTX describes subjective improvement in dyspnoea in 5 patients, no change in another 5 and worsening in 1 patient [32]. In our data, MTX was used in patients that resembled those of the never IS group except for higher mRSS and more frequent ground glass opacifications. Comparing MTX + GC to MTX alone displayed significant differences in mRSS, DLCO, FVC, FEV-1 and rates of ground glass opacifications. Nevertheless, its effect on the course of PFTs was negligible.

Overall, our data describe treatment patterns in patients with SSc-ILD that are used across European centres yet are only partially in accordance with EUSTAR recommendations. Most clinicians chose intensive IS in active lung disease. Common choices were not only CYC+/-GC as recommended by current guidelines, but also MMF + GC. None of the specific types of IS was clearly superior to another in influencing the course of lung function in any DLCO or FVC group. The only positive trends seen were in patients in the never IS group and patients taking GC, starting with > 75% of predicted FVC, and in the never IS group, patients taking AZA and MTX starting with > 75% of predicted DLCO all had a reduced rate of deterioration over time. Thus, if carefully monitored for changes in lung function, patients with SSc-ILD with only small PFT impairment might benefit from on-demand IS instead of ongoing IS.

Our study has some limitations: The retrospective design leaves us with some missing data, reducing the large number of patients within this data set to small groups when addressing specific questions of immunosuppressive treatment. Furthermore, changes in the prescription pattern might be missed, and data on when and why the immunosuppressive treatment was changed are lacking. Additional prospective data are urgently warranted. The prospective observational trial of the Seventh Framework Programme (FP7) project “DeSScipher” (a study to decipher the optimal management of systemic sclerosis) launched in 2012 will allow assessment of the dynamics of SSc-ILD-related treatment patterns in terms of escalation and de-escalation and to evaluate their efficacy.

## Conclusions

IS is broadly prescribed in SSc-ILD. Clusters of clinical and functional characteristics guide individualised treatment. The data favour differential decision-making pointing either to watchful waiting and close monitoring in the early stages or start of immunosuppressive treatment in patients with SSc-ILD and moderately impaired lung function. Advantages of specific IS are difficult to depict due to confounding by indication. Data do not support liberal use of GC in SSc-ILD.

## Additional file

Additional file 1: EUSTAR co-workers (DOCX 17 kb)

## Abbreviations

6MWD: Six minute walk distance
ACA: Anti-centromere antibodies
ACR: American College of Rheumatology
ANA: Anti-nuclear antibodies
a-TNF: Anti-tumour necrosis factor
AZA: Azathioprine
BMI: Body mass index
CI: Cardiac index
CK: Creatin kinase
CRP: C-Reactive protein
CYC: Cyclophosphamide
dcSSc: Diffuse cutaneous systemic sclerosis
DeSScipher: Study to decipher the optimal management of systemic sclerosis
DLCO: Diffusing capacity for carbon monoxide
D-PA: D-penicillamine
ESR: Erythrocyte sedimentation rate
EULAR: European League against Rheumatism
EUSTAR: European League against Rheumatism Scleroderma Trial and Research
FEV-1: Forced expiratory volume in one second
FP7: Seventh Framework Programme
FVC: Forced vital capacity
GC: Glucocorticoids
HRCT: High resolution computed tomography
ILD: Interstitial lung disease
IMA: Imatinib
IS: Immunosuppressive treatment
lSSc: Limited systemic sclerosis
LVEF: Left ventricular ejection fraction
MMF: Mycophenolate mofetil
mRSS: Modified Rodnan skin score
MTX: Methotrexate
NYHA: New York Heart Association
O2: Oxygen
PAPmean: Mean pulmonary arterial pressure
PAPsys: Systolic pulmonary arterial pressure
PFT: Pulmonary function test
PM SCL: Polymyositis scleroderma antibody
PVR: Pulmonary vascular resistance
PWP: Pulmonary wedge pressure
RNA: Ribonucleic acid antibody
RTX: Rituximab
RVSP: Right ventricular systolic pressure
SCL 70: Anti-topoisomerase I
SSc: Systemic sclerosis
TCZ: Tocilizumab
TLC: Total lung capacity
U1RNP: U1-small nuclear ribonucloprotein particle
VDAI: Valentini disease activity index
