# Supplementary material for: Systemic sclerosis associated interstitial lung disease - individualized immunosuppressive therapy and course of lung function: results of the EUSTAR group
Source: Arthritis Res Ther. 2018 Jan 30;20:17. doi: 10.1186/s13075-018-1517-z (PMC5791165; doi:10.1186/s13075-018-1517-z)
Supplement: Additional file 1: — EUSTAR co-workers (DOCX 17 kb) [file 13075_2018_1517_MOESM1_ESM.docx]

**EUSTAR Co-workers:** Otylia Kowal Bielecka, Bialystok, Poland. Jacob van Laar, Middlesbrough, UK. Carmen Pizzorni and Sabrina Paolino, Genova, Italy. Giovanni Lapadula, Bari, Italy. Radim Becvar, Prague, Czech Republic. Simona Rednic, Cluj-Napoca, Romania. Panayiotis Vlachoyiannopoulos, Athens, Greece. Veronica Codullo, Pavia, Italy. Jiri Stork, Prague, Czech Republic. Murat Inanc, Capa Istanbul, Turkey. Patricia E. Carreira, Cordoba, Spain. Srdan Novak, Rijeka, Croatia. Carlo Chizzolini, Geneva, Switzerland. Anna Kotulska, Katowice, Poland. Franco Cozzi, Padova, Italy. Blaz Rozman, Ljublijana, Slovenia. Armando Gabrielli, Ancona, Italy. Dominique Farge Bancel, Paris, France. Paolo Airo, Brescia, Italy. Roger Hesselstrand, Lund Sweden. Duska Martinovic, Split, Croatia. Alexandra Balbir-Gurman, Haifa, Israel. Nicolas Hunzelmann, Cologne, Germany. Raffaele Pellerito, Torino, Italy. Lisa Maria Bambara, Verona, Italy. Jadranka Morovic-Vergles, Zagreb, Croatia. Nemanja Damjanov, Belgrade, Serbia and Montenegro. Jörg Henes, Tübingen, Germany. Vera Ortiz Santamaria, Barcelona, Spain. Stefan Heitmann, Stuttgart, Germany. Dorota Krasowska, Lublin, Poland. Matthias Seidel, Bonn, Germany. Paul Hasler, Aarau, Switzerland. Harald Burkhardt, Frankfurt Main, Germany. Gianluigi Bajocchi, Reggio Emilia, Italy. José Antonio Pereira Da Silva, Coimbra, Portugal. Aleksandra Stankovic, Niska Banja, Serbia and Montenegro. Carlo Francesco Selmi, Milano, Italy. Mohammed Tikly, Johannesburg, South Africa. Ariane Herrick, Salford, UK. Raffaella Scorza, Milano, Italy. Francesco Puppo, Genova, Italy. Gitte Strauss, Hellerup, Denmark. Torhild Garen, Oslo, Norway. Eric Hachulla and David Launay, Lille, France. Guido Valesini, Roma, Italy. Ruxandra Maria Ionescu, Bucharest, Romania. Ana Maria Gherghe and Carina Mihai, Bukarest, Romania. Cord Sunderkötter, Münster, Germany. Jörg Distler, Erlangen, Germany. Pierluigi Meroni, Milano, Italy. Luc Mouthon, Paris, France. Vanessa Smith, Gent, Belgium. Francesco Paolo Cantatore, Foggia, Italy. Line Iversen, Copenhagen, Denmark. Carlos Alberto von Mühlen, Porto Allegre, Brazil. Maria Rosa Pozzi, Monza, Italy. Rüdiger Hein, Munich, Germany. Renata Sokoli, Wroclaw, Poland. Frédéric Hossiau, Brussels, Belgium. Juan Jose Alegre-Sancho, Valencia, Spain. Petra Saar, Frankfurt, Germany. Martin Aringer, Dresden, Germany. Rene Westhovens, Leuven, Belgium. Branimir Anic, Zagreb, Croatia. Maria Üprus, Tallin, Estonia. Sule Yavuz, Altunizade-Istanbul, Turkey. Brigitte Granel, Marseille, France. Carolina de Souza Müller, Curitiba, Brazil. Sergio Jimenez, Philadelphia, USA. Thierry Zenone, Valence, France. Simon Stebbings, Dunedin, New Zealand. Sarah Kahl, Bad Bramstedt, Germany. Vivien Hsu, New Brunswick, USA: François Spertini, Lausanne, Switzerland. Maura Couto, Viseu, Portugal. Itzhak Rosner, Haifa, Israel. Massimiliano Limonta, Bergamo, Italy. Nikolay A Mukhin, Moscow, Russia. Simon Stebbings, Dunedin, New Zealand. Alessandro Mathieu, Monserrato, Italy. Percival Sampaio-Barros, São Paolo, Brazil. Lisa Stamp, Christchurch, New Zealand. Douglas Veale, Dublin, Ireland. Kamal Solanki, Hamilton, New Zealand. Mengtao Li, Beijing, Chinal. Edoardo Rosato, Roma, Italy. Fahrettin Oksel, Bornova Izmir, Turkey. Cristina Mihaela Tanaseanu, Bucharest, Romania. Rosario Foti, Catania, Italy. Rodica Chirieac, Iasi, Romania. Cristiane Kayser, São Paolo, Brazil. Paloma García de la Peña Lefebvre, Madrid, Spain. Patrick Carpentier, Grenoble, France. Jacques Eric Gottenberg, Strasbourg, France. Ira Litinsky, Tel-Aviv, Israel. Algirdas Venalis, Vilnius, Lithuania. Lesley Ann Saketkoo, New Orleans, USA. Eduardo Kerzberg, Buenos Aires, Argentina. Dante Valdetaro Bianchi, Rio de Janeiro, Brazil.
